# Daylight-driven carbon exchange through a vertically structured microbial community

**DOI:** 10.3389/fmicb.2023.1139213

**Published:** 2023-05-26

**Authors:** James J. Moran, Hans C. Bernstein, Jennifer M. Mobberley, Allison M. Thompson, Young-Mo Kim, Karl L. Dana, Alexandra B. Cory, Steph Courtney, Ryan S. Renslow, James K. Fredrickson, Helen W. Kreuzer, Mary S. Lipton

**Affiliations:** ^1^Pacific Northwest National Laboratory, Richland, WA, United States; ^2^Department of Integrative Biology, Michigan State University, East Lansing, MI, United States; ^3^Department of Plant, Soil, and Microbial Sciences, Michigan State University, East Lansing, MI, United States; ^4^Faculty of Biosciences, Fisheries and Economics, UiT The Arctic University of Norway, Tromsø, Norway; ^5^ARC – The Arctic Centre for Sustainable Energy, UiT The Arctic University of Norway, Tromsø, Norway

**Keywords:** metaproteomics, stable isotope, cyanobacteria, microbial interactions, benthic microbial mat

## Abstract

Interactions between autotrophs and heterotrophs are central to carbon (C) exchange across trophic levels in essentially all ecosystems and metabolite exchange is a frequent mechanism for distributing C within spatially structured ecosystems. Yet, despite the importance of C exchange, the timescales at which fixed C is transferred in microbial communities is poorly understood. We employed a stable isotope tracer combined with spatially resolved isotope analysis to quantify photoautotrophic uptake of bicarbonate and track subsequent exchanges across a vertical depth gradient in a stratified microbial mat over a light-driven diel cycle. We observed that C mobility, both across the vertical strata and between taxa, was highest during periods of active photoautotrophy. Parallel experiments with ^13^C-labeled organic substrates (acetate and glucose) showed comparably less exchange of C within the mat. Metabolite analysis showed rapid incorporation of ^13^C into molecules that can both comprise a portion of the extracellular polymeric substances in the system and serve to transport C between photoautotrophs and heterotrophs. Stable isotope proteomic analysis revealed rapid C exchange between cyanobacterial and associated heterotrophic community members during the day with decreased exchange at night. We observed strong diel control on the spatial exchange of freshly fixed C within tightly interacting mat communities suggesting a rapid redistribution, both spatially and taxonomically, primarily during daylight periods.

## 1. Introduction

Microbes inhabiting phototrophic mat ecosystems are organized along steep geochemical gradients that are dynamically influenced by diel cycles which can themselves be associated with rapid shifts in geochemical (e.g., pH, redox, etc.) parameters linked to photosynthesis (Paerl et al., 2000; Visscher and Stolz, 2005; Ley et al., 2006; Harris et al., 2013; Al-Najjar et al., 2014; Bolhuis et al., 2014; Pages et al., 2014). Carbon (C) cycling in such environments is inherently complex due to close spatial positioning of otherwise disparate metabolic activities covering a range of phototrophic, lithotrophic, and heterotrophic processes (Wong et al., 2015). The resulting highly structured environments house diverse environmental conditions and microbial metabolisms over short distances which can lead to distal metabolic coupling between partners in different laminae. This is especially evident in C exchanges between photoautotrophs from oxic locations with anaerobes sequestered in anoxic zones of microbial mats (Canfield and Des Marais, 1991). Metabolites shared across environmental boundaries can take a wide range of forms including gas (e.g., O_2_, CO_2_, and CH_4_), dissolved aqueous phase compounds (e.g., sugar monomers, organic acids), and polymeric (e.g., extracellular polymeric substance; EPS) phases and the balance of fluxes between these C pools control the mat’s net biogeochemical footprint (Canfield and Des Marais, 1993; Dupraz and Visscher, 2005). While these spatially resolved C cycles have been characterized and compared between multiple mat ecosystems (Dupraz et al., 2009; Houghton et al., 2014; Stuart et al., 2016b), there remain fundamental gaps in our understanding of the timing and coordination of C exchanges between phototrophs and heterotrophs and the metabolic connectivity between different regions of microbial mats. Hot Lake is a hypersaline ecosystem in north central Washington, USA (Anderson, 1958) known for developing seasonal, benthic microbial mat where photoautotrophic processes are the primary C source (Lindemann et al., 2013). The mat communities in Hot Lake provide an opportunity for addressing questions related to C accumulation, exchange, and diel cycling within these complex structures.

Stable isotope tracers are a benchmark tool for investigating the fate of specific substrates through microbial ecosystems (Kelley et al., 2006; Van Der Meer et al., 2007; Bernstein et al., 2017b). Advances in laser ablation isotope ratio mass spectrometry (LA-IRMS) enable spatially resolved quantification of isotope tracer incorporation at sub-millimeter resolutions (Moran et al., 2011, 2014; Bernstein et al., 2017a). In this study, we traced C derived from ^13^C-bicarbonate into photoautotrophic and heterotrophic members of the Hot Lake microbial mat community and identified possible C transfer mechanisms. We used LA-IRMS to provide 50 μm spatial resolution of C distribution to compare C substrate uptake and redistribution from the various substrates in the context of the localized light regime within the mat. We then identified metabolites that contained ^13^C as candidates mediating interspecies C exchange, which were also identified as common constituents of extracellular polymeric substances (EPS). Finally, we demonstrated that substrate-derived C was incorporated into biomass of heterotrophic community members through metagenome-enabled, stable isotope-based proteomic analysis, and identified protein functional groups synthesized by the heterotrophs using this photoautotrophically fixed C.

## 2. Materials and methods

### 2.1. Site description

Hot Lake is an endorheic, hypersaline lake containing high levels of magnesium sulfate (i.e., epsomite) located in north central Washington State, USA (Jenkins, 1918). A combination of protection from strong winds (by the surrounding hillsides), small surface area, and geometry of the lake basin prevent mixing and give rise to a strong vertical salinity gradient through the water column (Anderson, 1958). Over the course of a summer season, the lower monimolimnion layer absorbs solar irradiation and, being prevented from releasing this heat by the overlying mixolimnion layer, can reach temperatures exceeding 60°C based only on solar heating and without any geothermal input (Zachara et al., 2016). During a previously recorded season having abnormally high precipitation inputs, the lake reached a surface area of 15,045 m^2^ and a total volume of 12,575 m^3^. Typical weather patterns produce high seasonal water levels (with lower total dissolved solids; TDS) in the spring/early summer and decreased water volumes (with higher TDS levels) later in the season (Zachara et al., 2016). The lake’s extreme salinity limits both aquatic vegetation and grazing pressure which enables seasonal assembly of a benthic microbial mat ecosystem present at a water depth/salinity band encircling the lake bottom; the mat presumably being limited below by extreme temperatures and constrained in upper growth by seasonal low water levels (Zachara et al., 2016). These mats are cyanobacteria dominated with an array of associated heterotrophic members and can reach multiple mm to roughly a cm in overall thickness (Lindemann et al., 2013; Brislawn et al., 2019). Previous sequence-based and stable isotope tracer explorations suggest microbial photoautotrophy constitutes a major source of C to the mat (Lindemann et al., 2013; Moran et al., 2014; Bernstein et al., 2017a). The reliance of this system on photoautotrophic input offers a unique opportunity to study the spatial dynamics of C cycling through a vertically structured microbial community.

### 2.2. Field sampling and incubations

On 31 July 2013, we harvested a portion of Hot Lake benthic microbial mat from ∼80 cm water depth (temperature of 43.8°C, total dissolved solids of 188 g/L) and immediately transferred the mat to shore for incubation. We incubated mat aliquots (∼15 cm diameter, round sections) in 1 L, Pyrex dishes in water collected at mat depth (∼80 cm) from the lake. Incubations were placed into a constructed Styrofoam incubator (Supplementary Figure 1) having an acrylic or solid lid to allow ambient solar irradiance or preclude light exposure. We directly added isotopically labeled (^13^C) and unlabeled (natural isotope abundance) substrates to the incubations. For the isotope labeling incubations, we added either 50% ^13^C sodium bicarbonate (made from 99% ^13^C, Sigma Aldrich, Lot # PR-22770) or unlabeled bicarbonate to a final concentration of 2.25 mM, unlabeled and labeled (uniformly ^13^C labeled, Icon Isotopes, Summit, NJ, USA, 99% ^13^C, lot CM-32113) glucose to a final added concentration of 5 mM, and unlabeled and labeled (uniformly ^13^C, 99%, Icon Isotopes, Summit, NJ, USA, Lot SM-93098) acetate to a final concentration of 2.2 mM such that each incubation contained one added substrate and unlabeled controls were provided for each substrate. We further included a ^13^C bicarbonate amended control which was incubated without light exposure. Incubation of the samples began at 11:30 a.m. local time. We manually opened or closed the acrylic cover on the incubation chamber to adjust the incubation temperature during the day and maintain it within a few degrees of the *in situ* temperature. The dark control required no venting for cooling. At night, a heated water bath was used to maintain temperature near *in situ* incubation temperature. Samples were harvested at eight time points during the incubation targeting key diel transition periods (dawn and dusk) and presumed periods of active photosynthetic activity (mid-day/high photon flux periods; Supplementary Figure 1). Irradiance data were collected at each time point using a Li-Cor 185 quantum/radiometer/photometer equipped with a LI-190 quantum sensor (Li-Cor, Lincoln, NE, USA) at the site of collection. We note that the thin acrylic sheet used to maintain mat near *in situ* temperature during daytime incubations had little impact on incident photosynthetically active radiation (PAR) which is the primary energy source supporting photoautotrophy in the mats being incubated. There is likely attenuation, however, of ultra-violet radiation by the acrylic cover during its intermittent use to help modulate incubation temperature. We further recognize that the Hot Lake water column (in part due to its high salinity) is able to attenuate incident visible light, including in portions of the PAR spectrum (Anderson, 1958). However, logistical constraints prevented incubation of the samples under a 0.8 m water column, meaning there was an increase in the incident light on the mat during the experiment relative to *in situ* growth.

Samples were collected by using a razor blade to cut and remove approximately 10 cm^2^ sections of the mat and efforts were taken to avoid removing the sides of the sample to minimize any artifacts from sunlight entering what would typically be the middle or lower portion of the mat cross section. Each sample was then immersed in lake water having no substrate amendments for a minimum of 30 min while being maintained in the dark. Previous work (Moran et al., 2014) demonstrated this approach was suitable for removing non-incorporated added substrate from the mat that would otherwise confound stable isotope measurement by LA-IRMS. Once rinsed in this way, samples were stored on dry ice for transport back to the laboratory and were then kept frozen (−20°C) until analysis. Samples from three time points were later subsection for proteomic and metabolomic analyses (those collected at dusk, dawn, and at the 24 h conclusion of the experiment).

### 2.3. Isotopic analysis

We performed LA-IRMS following the methods of Moran et al. (2014). Briefly, we lyophilized cross sections of the mat and then placed them into a custom-built laser ablation chamber that directly interfaced with an LSX-500 ablation system (Teledyne CETAC, Omaha, NE, USA) to target specific regions across the depth profile for analysis. We used a 50 μm sampling spot size with typically ∼200–300 μm between analysis spots and generated triplicate depth profiles, each being measured from the bottom to the top of the mat. We note that, due to the textured surface of the mat, all depth profiles were not equal in length. For this reason, we normalized the absolute distance measurements from each LA-IRMS depth profile on a scale of zero (upper surface of the mat) to one (bottom surface of the mat). We use this relative scale to report all LA-IRMS data.

Particulates resulting from each laser ablation sampling were entrained in a helium carrier gas and swept through a combustion reactor (nickel and platinum catalysts, 940°C, with a trace O_2_ addition) to convert all C to CO_2_ for isotope analysis. The CO_2_ was cryofocused in a liquid nitrogen trap then passed through a Nafion (Perma Pure, Lakewood, NJ, USA) drier before entering an IRMS for isotope measurement. Samples were analyzed on either a Thermo Delta V Plus (Thermo Fisher, Bremen, Germany) or Sercon 20–22 (Sercon, Crewe, Cheshire, England) IRMS. We used an in-house isotope standard (a standardized fishing line, δ^13^C = –27.71‰) to assess measurement accuracy and apply a measurement correction to the data. We report all isotope content in standard delta (δ) notation:


$$\delta^{13}\,C=\left(\frac{R\,s\,a\,m\,p\,l\,e}{R\,s\,t\,a\,n\,d\,a\,r\,d}-1\right)*1000$$

where R_*sample*_ is the measured ^13^C/^12^C ratio of a sample and R_*standard*_ is that of a sample. In this case we reference all isotope measurements to Vienna Pee Dee Belemnite (VPDB) with an R_*standard*_ of 0.0112372.

We analyzed the bulk (non-spatially resolved) δ^13^C of the samples using a Costech Analytical (Valencia, CA, USA) Elemental Analyzer (EA, ECS 4010 CHNSO Analyzer) coupled to a Thermo Scientific (Bremen, Germany) Delta V Plus IRMS. We lyophilized mat samples and homogenized them using a mortar and pestle prior to isotopic analysis. All samples were loaded into tin capsules for introduction to the EA. We maintained the EA combustion reactor (loaded with cobaltic oxide and chromium oxide catalyst) at 1,020°C and the reduction reactor (containing elemental copper) at 650°C. In-house glutamic acid isotope standards were calibrated against USGS 40 and USGS 41 (δ^13^C of −26.39 and + 37.63‰, respectively) and used as a basis for a 2-point data correction (Coplen et al., 2006).

We used acid rinsing/treatment of dried mat samples to remove acid-labile components. Briefly, we applied 5 ml of 1.2 N HCl and allowed this to react with the dried, homogenized mat (typically 0.5 to 1 g) overnight. Following this extraction, we tested the pH to ensure the acid had not been fully consumed. We then centrifuged the samples (5000 rpm, 20 min) and decanted off the aqueous layer. We rinsed the resulting pellets three times with 10 ml deionized water (with shaking for 3 min) using centrifugation and decanting to remove each wash. Finally, the samples were dried prior to weighing and isotopic analysis via EA-IRMS.

### 2.4. Estimating C replacement in biomass

We used a comparison of isotope data from experimental and control incubations to quantify the conversion of substrate-derived (bicarbonate, acetate, or glucose) C into biomass across the mat depth profiles measured using LA-IRMS over the experimental time series. We applied an isotopic mass balance approach based on the fractional abundance of ^13^C in the mat biomass incubated with a ^13^C labeled substrate, control biomass incubated with a substrate not containing ^13^C, and the added substrate (see Supplementary Information).

### 2.5. Proteomic analysis

Proteins were extracted using methanol-chloroform method as described previously (Folch et al., 1957). Proteins were denatured and reduced under the following conditions: 8M Urea, 5 mM DTT, 100 mM ammonium bicarbonate buffer at 60°C for 30 min. After denaturing, samples were diluted eightfold with 100 mM ammonium bicarbonate and a sufficient amount of calcium chloride was added to achieve 1 mM. Tryptic digestion was performed for 3 h at 37°C with 1:50 (w/w) trypsin-to-protein ratio. The digested sample was desalted and cleaned via solid phase extraction (SPE) C18 (Supelco, Bellefonte, PA, USA). Sample was concentrated in Speed-Vac (Thermo Savant, Holbrook, NY, USA) before performing BCA Assay to determine final peptide concentration.

We performed LC-MS sample analyses using a Waters nanoEquity™ HPLC system (Waters, Milford, MA, USA) coupled to a Q Exactive (Thermo Scientific, San Jose, CA, USA) mass spectrometer. LC pumps were set up to load the sample first on a solid phase extraction (SPE) column and then separated on a reversed-phase analytical column. Reversed-phase analytical capillary columns were made in-house by slurry packing 3-μm Jupiter C18 stationary phase (Phenomenex, Torrence, CA, USA) into a 70-cm length of 360 μm o.d. × 75 μm i.d. fused silica capillary tubing (Polymicro Technologies Inc., Phoenix, AZ, USA). The SPE column (360 μm o.d. × 150 μm i.d.) of 5 cm length was similarly made with 3.6-μm Aeries C18 particles. Mobile phases consisted of 0.1% formic acid in water (mp A) and 0.1% formic acid in acetonitrile (mp B). Sample was loaded on the SPE column via a 5 μL sample loop for 30 min and then separated by the analytical column using a 215 min gradient from 99 to 25% A at a flow rate of 0.3 μL/min. Mass spectrometry analysis was started 30 min after the sample was moved to the analytical column and lasted for 180 min.

The LC column interfaced with a Q Exactive hybrid quadrupole/Orbitrap mass spectrometer via an electrospray emitter for the ionization of effluent and introduction to the mass spectrometer that was operated in data-dependent analysis mode. A spray voltage of 2.2 kV was used for electrospray ionization and inlet capillary to the mass spectrometer was maintained at a temperature of 325°C for ion de-solvation. A primary survey scan was performed in the mass range of 300 to 1800 Da at a resolution of 70,000 (defined at mass 200) and AGC (automatic gain control) setting of 5 × 10^6^ ions. The top 15 ions from the survey scan were selected by a quadrupole mass filter and fragmented by high energy collision dissociation (HCD) in collision with nitrogen and mass analyzed by the Orbitrap at a resolution of 35,000. An isolation window of 2 Da was used for the isolation of ions and collision energy of 28% and for HCD with AGC setting of 1 × 10^5^ ions. Mass spectra were collected for 180 min. The mass spectrometry proteomics data have been deposited to the ProteomeXchange Consortium via the PRIDE (Deutsch et al., 2017) partner repository with the dataset identifier PXD012190 and 10.6019/PXD012190.

### 2.6. Proteomic data analysis

Mass spectra data were searched against a protein database constructed from draft genomes assembled from the laminar metagenome constructed from individual cross-sections of the Hot Lake microbial mat (Mobberley et al., 2017). The reconstructed draft genomes used can be found at https://github.com/jenmobberley/lamMG_assembly. The MS-GF + search algorithm was used to match tandem mass spectrometry (MS/MS) spectra to peptide sequences (Kim et al., 2008). Partial tryptic cleavage, dynamic modification of methionine oxidations, and maximum 20 ppm parent ion mass tolerance were included in the search. Peptide-spectrum matches were filtered to a 1% FDR (Ma et al., 2009; Tabb et al., 2009). We identified specific peptides containing labeled C using the Stable Isotope Probing Protein Extraction Resource (SIPPER) software (Slysz et al., 2014). Unlabeled bicarbonate samples were used as a comparison set for each of the labeled acetate, glucose, and bicarbonate sets. The filtering cutoffs used were for a false positive cutoff near 5% with the following parameters as guidelines: Fit Score Labeled (< = 0.8), I Score (< = 0.6), Sum of Ratios (> = 0), Contig Score (> = 0), Percent Incorporation (> = 0.5), and Percent Peptide (> = 0.5), as described by Slysz et al. (2014) for tight filtering. We attributed both labeled and unlabeled peptides to proteins from two large taxonomic pools, the dominant cyanobacteria and the dominant non-cyanobacterial community members (Mobberley et al., 2017), and weighted the resulting labeled vs. unlabeled peptide distribution based on spectra abundance of each peptide. Counts of labeled protein are presented as relative abundance of total proteins observed for each time point and incubation condition. To broadly characterize the function of labeled proteins, we used the Kyoto Encyclopedia of Genes and Genomes (KEGG) ontology to assign proteins to metabolic pathways (Kanehisa et al., 2015). Due to some proteins being used in multiple metabolisms, single proteins may be counted multiple times. Heatmaps of the relative abundance of labeled proteins assigned to the KEGG metabolic pathways were created in R using pheatmap (Kolde, 2015) with relative abundances normalized by square-root transformations. This approach was not designed to assess the extent of labeling (number of ^13^C molecules) within each peptide but focused on characterizing the number of distinct proteins to which labeled substrate-derived C was disseminated. This approach can provide a more taxonomically specific measure of metabolic activity than measuring incorporation into fatty acids (Jehmlich et al., 2009; Von Bergen et al., 2013).

### 2.7. Nuclear magnetic resonance microimaging

We used nuclear magnetic resonance microimaging and localized spectroscopy to assess the transport properties of the Hot Lake mat and monitor acetate penetration into the mat over time. Similar to Renslow et al. (2010, 2013), NMR experiments were performed at 500 MHz for protons (^1^H) using a Bruker Avance III imaging spectrometer (Bruker Instruments, Billerica, MA, USA) with an 11.7 T, 89 mm vertical bore, actively shielded superconducting magnet. The mat sample was collected and degassed inside a 15-mm NMR tube and interrogated using a Bruker microimaging gradient insert (Micro2.5). ParaVision v5.1 imaging software (Bruker Biospin, Billerica, MA, USA) was used to collect the data. Data processing was performed using custom written Python scripts. Measurements included: (1) rapid multidirectional magnetic resonance imaging (MRI) to verify correct sample positioning; (2) 2D Fourier transform (2DFT) MRI; (3) diffusion tensor imaging for generating diffusion coefficient profiles (DtiStandard); (4) localized magnetic resonance spectroscopy (PRESS), and (5) and chemical shift selective imaging for generating porosity profiles (mic_chess). For further details, please see section “2. Materials and methods” and Supplementary material in Lone et al. (2015).

### 2.8. Metabolite analysis

Metabolites were extracted from the microbial mat samples using chloroform/methanol mixture (Cole et al., 2014). The solvent mixture was added to 50 mg of wet microbial mat samples that was subsequently homogenized with glass beads while the metabolite extraction was performed. Supernatant was collected after centrifugation (15,000 × *g* × 5 min at 4°C) and 100 μL of each aqueous layer (water + methanol), containing hydrophilic metabolites, was transferred to a new clean vial, dried under a speed-vacuum concentrator then stored in the −70°C freezer until the instrumental analysis, at which point they were chemically derivatized as previously reported prior to analysis (Kim et al., 2015). Briefly, the extract metabolites were derivatized by methoxyamination and trimethylsilylation (TMS), then the samples were analyzed by GC-MS using an Agilent 7890A gas chromatography system coupled with a single quadrupole 5975C mass spectrometer (Agilent Technologies, Inc., Santa Clara, CA, USA) equipped with an HP-5MS column (30 m × 0.25 mm × 0.25 μm) with data collection from 50 to 550 m/z. We maintained an injector temperature of 250°C, performed a splitless injection, and ramped the GC oven from 60 (1 min hold) to 325°C at 10°C/min with a final hold of 5 min. The raw GC-MS data were analyzed by manually for getting mass isotopomer distribution of selected metabolites, and they were compared with the control samples (^12^C bicarbonate) for each time point to identify ^13^C labeled analytes.

## 3. Results

### 3.1. Stable isotope analysis

**FIGURE 1:**
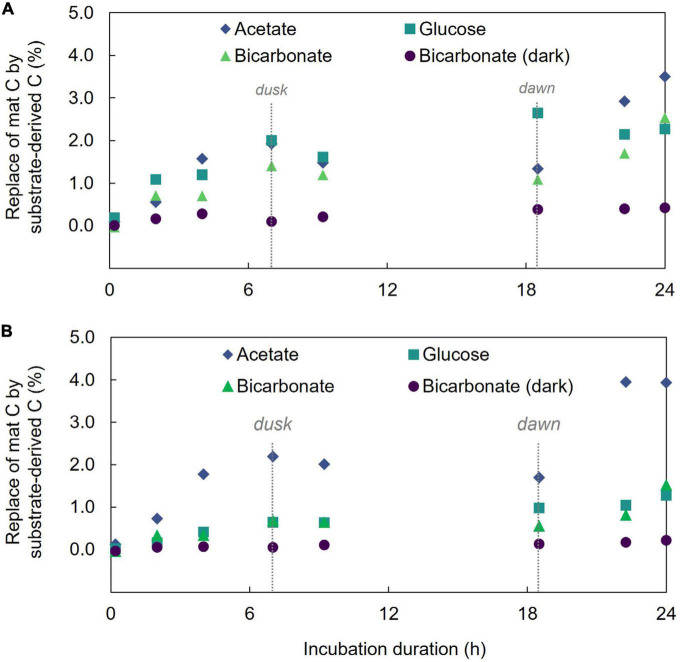
Incorporation of ^13^C into microbial mat community and acid-rinsed mat. **(A)** The percent replacement of mat bulk C by that derived from the added substrate. **(B)** The percent replacement of mat C by substrate derived C in the acid-treated mat.

Isotope (δ^13^C) analysis of bulk mat samples showed increasing ^13^C content over the 24 h of incubation in each of the substrate incubations which demonstrates incorporation of substrate-derived carbon into mat material (Figure 1 and Supplementary Table 1). Far less ^13^C was incorporated in ^13^C-bicarbonate incubations maintained in the dark (at 24 h, light incubation had δ^13^C = 187.3 ± 3.2‰ and dark incubation had δ^13^C = 30.83 ± 1.2‰; two-sample student’s *T*-test *p* = 3.7E-18) which highlights the light-driven nature of the mat’s autotrophic activity, dominance of photoautotrophy over chemolithoautotrophy in this mat during the investigation, and is consistent with previous results (Moran et al., 2014). Additionally, δ^13^C enrichment was not observed in control incubations devoid of a ^13^C tracer (indicating no cross contamination between incubations).

Hot Lake microbial mat samples were previously demonstrated to contain the carbonate materials including magnesite, calcite, and aragonite (Zachara et al., 2016), and at least some of the ^13^C bicarbonate might be expected to accumulate in these minerals. Microbial mats are typically characterized by the presence of EPS that provide a matrix to support the physical structure and integrity of the mat, presenting another mat component in which ^13^C might be incorporated. Compounds composing EPS including carbohydrates, amino acids, and some proteins can be removed from underlying microbial biomass by gentle washing in dilute acid (Ng et al., 2007; Brodie et al., 2011) which can also remove carbonate from a sample. To begin to trace ^13^C incorporation from the three different substrates in the mats over time, we performed a gentle acid washing treatment followed by δ^13^C measurement in the samples to remove any inorganic C contained within the mat as well as the EPS components. Results (Figure 1) show divergent trends between substrate incubations. More specifically, acid treatment decreased the bulk ^13^C enrichment signal in the bicarbonate and glucose incubations but increased the signal in the acetate incubation. The decrease in ^13^C signal with the acid treatment suggests that the EPS and carbonate phases, on average, contained a higher proportion of ^13^C tracer than the remaining material (i.e., cellular biomass) in the samples incubated with labeled bicarbonate or glucose. Conversely, in the acetate incubations the increase in δ^13^C following the acid treatment suggests the removed material contained less tracer-derived-C than the cellular biomass.

To test whether the organic material removed (presumably EPS components) by the acid treatment was ^13^C-enriched, we performed a follow up analysis on a limited subset of remaining samples. In addition to the pre- and post-acid treatment analyses we also collected the aqueous supernatant from the acid treatments, dried down this material, and evaluated its δ^13^C. Since all carbonate-derived material in the acid treatment would be converted to CO_2_ and purged from solution by the low pH in the acid washing, the collected supernatant would be comprised of only aqueous organic material that was removed during the treatment. The results (Supplementary Table 2) confirmed that this removed aqueous material was more strongly enriched in ^13^C than the mat biomass in the bicarbonate and glucose incubations but not under the acetate conditions.

**FIGURE 2:**
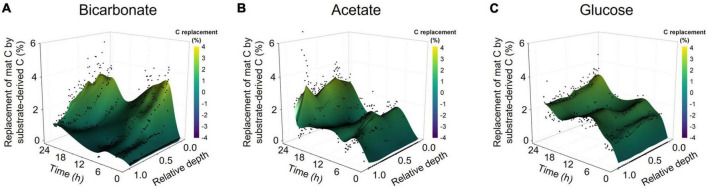
Spatially resolved accumulation of C from diverse substrates into microbial mat. Laser ablation isotope ratio mass spectrometry (LA-IRMS) was used to spatially resolve and quantify incorporation of ^13^C labeled substrates into normalized vertical profiles (zero being the upper surface and one being the bottom) of microbial mat over a diel time series in the **(A)** bicarbonate, **(B)** acetate, and **(C)** glucose incubations.

The bulk isotope analyses quantified incorporation of substrate-derived C into the mat biomass but did not provide vertical localization of freshly incorporated C with mat structure. LA-IRMS was used to evaluate the localization of substrate-derived C incorporation across the approximately 1 cm thick mat in 8 timepoint subsamples collected over the 24 h incubation. Our analysis showed the bulk of inorganic C incorporation (from bicarbonate) occurred near the surface of the mat, with diminishing amounts of ^13^C detected with increasing depth (Figure 2A). In contrast, the incorporation of ^13^C from acetate and glucose revealed rapid uptake of label throughout the depth profile without strong vertical localization (Figures 2B, C and Supplementary Table 3) despite presumed changes in dominant organisms and geochemical conditions down the depth profile. This depth-independent labeling pattern was observed from the earliest time point (20 min of incubation) and indicates active heterotrophic metabolism throughout the mat. The incorporation of ^13^C from acetate and glucose throughout the mat depth profile at even the earliest time points also highlights the rapid diffusion of these substrates into the mat, indicating that mass transfer limitation was not a major factor within the experimental time scales observed. Hence, the differences observed between C-transfer across depth profiles were due to differing rates of substrate uptake and metabolite exchange. To corroborate this, we tracked applied acetate in a mat sample using nuclear magnetic resonance (NMR) microimaging and observed rapid penetration from the top to the bottom of the mat (Supplementary Figure 2).

We also used LA-IRMS measurements to quantify the uptake and vertical redistribution of ^13^C derived from either labeled bicarbonate, acetate, or glucose by the community over the 24 h diel to identify the initial localization of substrate uptake and subsequent mobility of the C. At the conclusion of the experiment, the average amount of biomass C replaced by ^13^C from either bicarbonate, acetate, or glucose substrate as measured by LA-IRMS was not significantly different (single factor ANOVA, *p* = 0.1426) indicating similar overall C uptake: 2.46% (stan. dev. 0.21%), 2.19% (stan. dev. 0.07%), and 2.39% (stan. dev. 0.13%) replacement of mat C with C from the respective substrate. For all the substrates, label uptake increased during the day followed by a partial loss of this C under dark conditions (Figure 1), but the amount of C loss appeared higher for inorganic substrate (44% of C derived from freshly fixed bicarbonate was lost between timepoints 4 and 6) than for either of the organic substrates (14%, and 12% of freshly fixed acetate-, or glucose-derived C, respectively).

**FIGURE 3:**
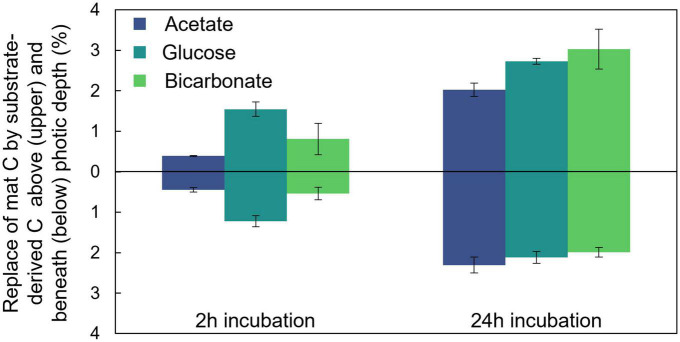
Distribution of fixed carbon below the photic depth. A portion of C assimilated from each of the added substrates was located both within and below the photic depth in the mat as depicted here after both 2 and 24 h of incubation. Errors bars correlate to one standard deviation of triplicate measurements.

To explore the partitioning of bicarbonate-derived C across the photic-aphotic boundary we assumed that a scalar irradiance ≤10 μmol photons PAR m^–2^ s^–1^ defined the boundary condition between these zones (Supplementary Figure 1C; Bernstein et al., 2017a) and determined the ratio of average % C replacement by the added labeled substrates across this boundary (Figure 3). At both timepoints examined (2 and 24 h) we observed nearly equal (42.7 and 40.2%, respectively; two-sample student’s *T*-test *p* = 0.41) proportions of the fixed, bicarbonate-derived C was positioned below the photic depth despite a substantial increase in the overall amount of fixed C in the mat. After 24 h, the average amounts (per cents) of photic zone C replaced by glucose- and bicarbonate- derived C were statistically indistinguishable, while the amount replaced by acetate was lower (Tukey’s HSD, *p* < 0.05). After 24 h, the average replacement in the aphotic zone was statistically indistinguishable for the three substrates (single factor ANOVA, *p* = 0.11).

### 3.2. Metabolite analysis

We traced ^13^C derived from each of the three substrates into six prominent metabolites identified in the samples: glucose, sucrose, trehalose, glucosylglycerol, 3-hydroxybutanoate, and 3-hydroxypentanoate (Table 1 and Supplementary Figure 3). Incorporation of ^13^C from glucose was detected in all the selected metabolites at all-time points, though residual substrate that was not completely removed during sample washing would contribute to the detection of labeled glucose in these samples. Incorporation of ^13^C derived from bicarbonate was readily detected in glucose, sucrose and trehalose, metabolites associated with the synthesis and maintenance of compatible solutes, by the dusk timepoint (Time 4, Table 1 and Supplementary Figure 3), indicating active biosynthesis during sunlight hours. The degree of labeling in these metabolites was noticeably reduced at the dawn time point (Time 6), suggesting both that fixing of inorganic C into these compounds occurs in daylight, consistent with photoautotrophic processes, and that these metabolites undergo rapid turnover. No incorporation of label from bicarbonate into the two carboxylic acids was detectable at dusk, with minimal incorporation evident by the following noon (Time 8).

**TABLE 1:** Incorporation of ^13^C from glucose, bicarbonate, or acetate into metabolites over a diel cycle.

		Measured metabolite																	
		Glucose			Sucrose			Trehalose			Glucosyl-glycerol			3-hydroxy-butanoate			3-hydroxy-pentanoate		
	Timepoint*	4	6	8	4	6	8	4	6	8	4	6	8	4	6	8	4	6	8
Incubation substrate																			
	Control	−	−	−	−	−	−	−	−	−	−	−	−	−	−	−	−	−	−
	Bicarbonate	+	+/−	+	+	+/−	+	+	−	−	+	+/−	+	−	−	+/−	−	−	+/−
	Acetate	+	−	+	+	+	+	+	−	+	+	+	+	−	+	+	−	+	+
	Glucose	+	+	+	+	+	+	+	+	+	+	+	+	+	+	+	+	+	+

*4 = dusk; 6 = dawn; 8 = solar noon of second day.

The label incorporation patterns in the ^13^C-acetate incubations varied by metabolite. Within these incubations, ^13^C was readily detected in glucose, sucrose, trehalose, and glucosylglycerol at dusk (Time 4). The degree of labeling of glucose and trehalose was diminished at dawn (Time 6) but increased by the following noon (Time 8). Incorporation from acetate into sucrose and glucosylglycerol increased throughout the time series with no diminishment at dawn. Incorporation into the 3-hydroxybutanoic and 3-hydroxypentanoic acids was negligible at dusk, but strongly evident at dawn and increased further in the noon time point.

### 3.3. Proteomic analysis

Isotopically labeled peptides, defined where the mass spectrum visualized at the apex of a peptide’s elution showed a clear isotope labeling pattern and exhibited no evidence of co-eluting peptides (Slysz et al., 2014), were mapped to reconstructed genomes of cyanobacterial and heterotrophic members of the Hot Lake ecosystem, and protein identification was inferred from peptide-spectrum matches using standard methods (Mobberley et al., 2017). We clustered the identified taxa into two groups (cyanobacterial and non-cyanobacterial proteins) to represent the photoautotrophic and heterotrophic members of the community.

**FIGURE 4:**
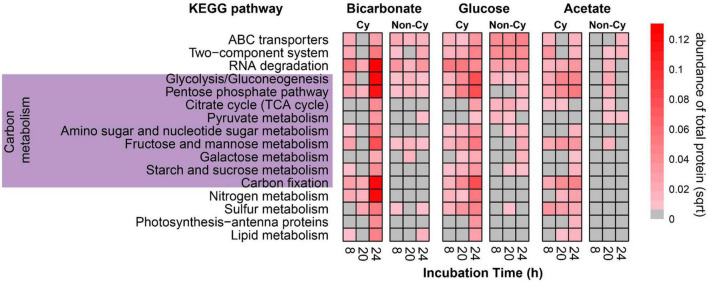
Selected metabolic pathways showing active incorporation of ^13^C label into proteins of cyanobacterial (Cy) and non-cyanobacterial (Non-Cy) community members across a 24 h labeling experiment. This heatmap represents the relative abundance of labeled protein, square-root transformed, that can be functionally assigned to representative broad metabolic processes. The protein assignments were based on KEGG ontology and single proteins may be represented multiple times due to functions associated with multiple pathways. Gray fill indicates no labeled proteins for this pathway were detected.

In total, 5.6, 1.5, and 5.9% of the proteins in respective bicarbonate, acetate, and glucose incubations were labeled after 24 h (Supplementary Table 4). Proteins associated with both cyanobacteria and heterotrophic community members were labeled, indicating incorporation of label into biomass through both photoautotrophic and heterotrophic processes (Figure 4). Interestingly, for all substrates (both inorganic and organic) and at all-time points, we observed a higher number of labeled proteins associated with cyanobacterial vs. heterotrophic (non-cyanobacterial) community members. This difference could arise from several mechanisms including basal uptake of non-^13^C labeled, dissolved organic C compounds present in Hot Lake water by heterotrophs, reduced protein synthesis rates in the heterotrophic organisms, or a higher overall biomass associated with cyanobacteria members of the system vs. the associated heterotrophic community.

The number of labeled proteins in the cyanobacteria diminished overnight and increased strongly the following day, consistent with active carbon fixation and protein synthesis during daylight hours and indicating rapid turnover of at least some of the newly synthesized proteins. In contrast, the range of labeled proteins in the heterotrophs increased over the time course, consistent with increasing transfer of labeled photosynthate to the heterotrophic community members. Labeled proteins in both autotrophs and heterotrophs included those involved in substrate transport (ABC transporters), basic energy metabolism, and sugar catabolism (Figure 4), although no proteins involved with starch or sucrose synthesis were labeled in the heterotrophs. Labeled proteins associated with photosynthesis, C fixation, and N metabolism were detected only in the autotrophs.

The incorporation of C derived from acetate and glucose into proteins was not strongly influenced by the diel cycle; we observed an increase in the distribution of substrate-derived C into protein biomass of both autotrophs and heterotrophs at each time point (Figure 4). This increase is consistent with direct assimilation of acetate and glucose derived C into amino acids without regard to light availability or associated redox variations within the system. ^13^C derived from glucose was incorporated at higher levels and into more proteins than was ^13^C from acetate or bicarbonate (Figure 3). We observed the strongest time-dependent incorporation of glucose-derived C into cyanobacterial proteins involved in energy production and C and nutrient cycling. The heterotrophic members of the community were actively using glucose as a C source as evidenced by incorporation into enzymes involved in environmental sensing apparatus, substrate transport, and energy production. The incorporation of C derived from acetate, an important precursor in energy generation and fatty acid biosynthesis, into proteins was not as high as that from glucose or bicarbonate (Figure 4).

## 4. Discussion

### 4.1. Mat-scale C uptake dynamics

The bulk isotope analysis of mat and acid-washed mat samples revealed uptake of the applied substrates in even the shortest measured timepoint (0.2 h). Further, the data derived from acid-washed samples suggest formation of EPS within this timeframe, which indicates rapid biological uptake, chemical transformation, and extracellular placement. Previous work by Gonzalez-Nayeck et al. (2022) in phototroph-dominated stratified communities also showed rapid conversion of dissolved inorganic carbon into glucose-rich EPS material which then served as a common carbon source for mat biosynthesis. Similar, high rates of exchange between bicarbonate and glucose in the Hot Lake mat may help explain the similar behavior between the applied bicarbonate and glucose during daylight periods of the incubation. The relatively lower proportion of acetate-derived C being converted to EPS materials could be due to either the organisms in the community that synthesize EPS being distinct from those that take up acetate, or acetate is subjected to metabolic routing that does not include EPS synthesis. This observation is consistent with previous reports of acetate being preferentially converted to lipids (as opposed to bicarbonate, glucose, and formate) which was presumably driven by the biochemical efficiency of direct incorporation of acetate into acetyl-CoA used for lipid synthesis (Schubotz et al., 2015). Thus, the bulk isotope results are consistent with a shift in biochemical fate for acetate in the mat vs. glucose and bicarbonate.

The metabolite analysis revealed distinct chemical transformations with different rates and diel dependencies for the three labeled substrates. Applied glucose was rapidly converted to each of the six metabolites tracked during the incubation and showed no diel dependence. Acetate transformations, however, occurred more slowly and displayed some diel dependence, consistent with a presumed shunt of acetate-derived C to synthesis of lipids or other cellular components (acetate being converted to cellular components is supported by the data from the acid washing experiments that showed increased ^13^C content in samples from which inorganic C and EPS was removed). Further, the diminishment in the incorporation of ^13^C from acetate into glucose overnight could indicate that the reactions are occurring in phototrophs and, therefore, are light-dependent. An alternative explanation is that this metabolic processing is O_2_ dependent. During daylight when photosynthesis is active, the upper regions of the mat are oxygenated even if the lower laminae might remain anoxic; however, at night when photosynthesis ceases, the mat becomes anoxic throughout its vertical profile (Bernstein et al., 2017a). Our current data do not allow us to distinguish between these two possibilities. The bicarbonate incubations showed strong conversion to glucose, sucrose, trehalose, and glucosyl-glycerol during daylight hours with these abundances decreasing overnight. Interestingly, these compounds are common osmolytes in hypersaline cyanobacterial systems (Oren, 2015) so their accumulation may represent a response to the ongoing salinity stress in this hypersaline environment. The decrease in the abundance of these compounds overnight is consistent with rapid chemical turnover which may signify a pathway for autotroph to heterotroph C exchange.

The LA-IRMS results showed similar overall C uptake for each of the applied substrates, but different levels of night-associated C loss. This diurnal loss of newly acquired C was consistent with previously reported observations from related mat ecosystems where C usage by a combination of nighttime fermentation and sulfate reduction offset a portion of daily photoautotrophic C fixation (Burow et al., 2013; Houghton et al., 2014; Lee et al., 2014; Stuart et al., 2016b). Previous related studies largely reported this diel C cycling qualitatively and did not quantify net C uptake and remineralization. An exception to this was presented by Lee et al. (2014) who applied an electron balance to estimate a loss of ∼81% of freshly fixed C to fermentation reactions at night in a hypersaline phototrophic mat system. To our knowledge, our result represents the first direct measurement and quantification of C uptake and remineralization over a diel cycle in a microbial mat ecosystem.

### 4.2. Transfer of photoautotrophically fixed C to aphotic zone

Incorporation of the organic substrates was well distributed through the mat thickness and highlighted distribution of heterotrophs throughout the mat profile. Previous work revealed cyanobacteria could be found through the mat’s layers but tended to be more abundant in the upper reaches of the profile in areas of higher light intensity, while anoxygenic phototrophs were more evenly distributed over the mat thickness (Mobberley et al., 2017). In our incubations, photoautotrophically fixed C (derived from bicarbonate) was concentrated at the surface of the mat with rapid decrease in concentration below the top mm and negligible incorporation into the bottom lamina at early time points. Note, our work does not preclude the occurrence of chemolithoautotrophy, anoxygenic phototrophy, or anapleurotic processes as mechanisms for biologic incorporation of inorganic C. However, the data collected from a dark control incubation indicates the vast majority of the observed autotrophy is light-dependent, so our discussion of the resulting data focuses on photoautotrophy. The decrease in inorganic C accumulation (at early timepoints) with increased depth was likely controlled by light penetration into the mat, which limited photoautotrophy to fairly short distances (∼ 1 mm) below the surface (Jørgensen and Des Marais, 1986; Al-Najjar et al., 2010; Bernstein et al., 2016; Mobberley et al., 2017).

To explore the rate and amount of transfer of photoautotrophically fixed C to zones of the mat where photoautotrophy could not take place, we compared the amount of substrate incorporation into biomass in the light (photic zone) and underlying dark (aphotic zone) sections of the mat (Figure 3). This analysis revealed a rapid (within 2 h) transfer of photoautotrophically fixed C to aphotic regions of the mat. In addition, results revealed proportional bicarbonate-derived C in the photic zone in early (2 h) and late (24 h) timepoints despite an overall increase in the total amount of bicarbonate-derived C in the mat. It is well established that autotrophic processes play a central step in microbial carbon supply in laminated microbial mat communities (Franks and Stolz, 2009) so it was not surprising to observe photoautotrophically fixed carbon in the lower reaches of the vertical mat profile. The low ^13^C accumulation in mat biomass in the dark incubations demonstrated the dominant role of photoautotrophy (compared to the combined chemolithoautotrophy and anapleurotic fixation) in providing carbon for the system. What is potentially surprising, however, is the temporal connectivity in carbon exchange between the photic (initial site of fixation) and aphotic (by definition, not able to support photoautotrophy) zones. The similar amount of C replacement by glucose and bicarbonate after 24 h of incubation highlights the potential interplay between small, bioavailable organic compounds (such as glucose) produced and released into the mat from photoautotrophy and the carbon present in total mat biomass where, for example, extracellular glucose can be a carbon source for biomass production even for the primary photoautotrophs in the system (Stuart et al., 2016b). Further, the degree of carbon exchange between physiological groups (e.g., photoautotrophs and heterotrophs) is so fluid in mat systems that it can blur traditional markers of trophic level distinctions found in stable isotope evaluation (Gonzalez-Nayeck et al., 2022). This rapid exchange of C may offer one potential mechanism for explaining the indistinguishable proportion of substrate-derived C in biomass within aphotic zone across all incubation types after 24 h. Thus, despite the requirement for light, photoautotrophy provided fresh C to aphotic zones of the mat as effectively as the exogenously added substrates supported heterotrophic uptake under our experimental conditions.

### 4.3. Incorporation of label into mat biomass

Our bulk stable isotope ratio measurements of washed mat biomass demonstrated that ^13^C from all the labeled substrates was incorporated into biomass. It did not, however, establish definitively that photoautotrophically fixed C was incorporated into the biomass of the heterotrophic members of the community. An alternative hypothesis to explain the presence of ^13^C below the photic zone was that the EPS synthesized by the autotrophs permeated the mat, or that this EPS, and not the biomass of heterotrophic organisms, accounted for our detection of ^13^C in the aphotic zone of the mat (despite presumed loss of this material during the acid washing). We turned, therefore, to metaproteomics to help assess C exchange between autotrophic and heterotrophic community members. We note that cyanobacteria can functionally serve as heterotrophs during night portions of the diel but, for the metaproteomic analyses, distinguish cyanobacterial proteins as associated with the primary photoautotrophic mat members and other members as predominantly functional heterotrophs (consistent with previous work; Lindemann et al., 2013).

The results from the metaproteomic analysis of samples labeled with bicarbonate show that proteins from both cyanobacteria and mat heterotrophs were labeled within 8 h (Figure 4). Such rapid integration of substrate and conversion of this C to proteins is not unheard of and has been previously demonstrated in similar studies of photoautotrophic mats (Steinke et al., 2020; Ataeian et al., 2022). Collectively, our results show that fixed inorganic C is rapidly shared—on the time scale of less than 8 h—between cyanobacteria and heterotrophic populations and highlights the degree of metabolic coupling and rate of carbon exchange throughout the mat system. The proteomic data further demonstrates incorporation of acetate- and glucose- derived C into heterotrophic biomass at each timepoint across the diel cycle and did not reveal shifts in substrate conversion to proteins based on presumed diel redox variation and nighttime shift to fermentation metabolisms. This is consistent with fermentation patterns reported in a separate hypersaline mat where cyanobacteria remained metabolically active at night through fermentation of daytime-derived photoautotrophy products (Lee et al., 2014). The cyanobacterial enzymes detected containing acetate-derived C were similar to those for glucose, indicating low molecular weight organic C has the potential to be rapidly metabolized by photoautotrophic primary producers. Previous work suggests cyanobacteria can target EPS as a nutrient resource during periods of active photosynthesis as well as a carbon resource during dark periods of the diel (Stuart et al., 2016a) and our data are consistent with similar processes occurring here.

### 4.4. Potential mechanisms of C transfer from autotrophs to heterotrophs

**FIGURE 5:**
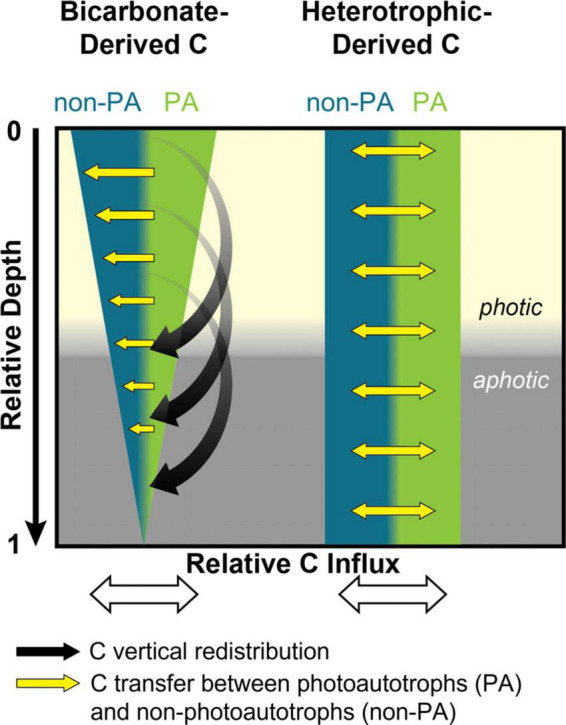
Model for movement of carbon through the Hot Lake mat. Distinct patterns of C propagation through mat biomass are depicted based on their metabolic process (i.e., photoautotrophic or heterotrophic) of entry into the mat.

Our data clearly show that freshly fixed C is transferred from the photic zone to the aphotic zone of the mat while also being exchanged between autotrophs and heterotrophs (Figure 5). Previously identified mechanisms of C exchange from the photic to aphotic zone include cell migration or transfer of fixed C through exchange of metabolites, EPS components, or necromass. We have shown that freshly fixed inorganic C is rapidly incorporated into both soluble metabolites and EPS components, and these substances could therefore provide a means of C transfer. Cell migration over a diel cycle is well documented in comparable microbial mat systems (Garcia-Pichel et al., 1994; Villanueva et al., 2007; Dillon et al., 2009). However, the known distance for cell migration over diel cycles is fairly well constrained to less than 1 mm. At the time of our experiments, the Hot Lake mat was approximately 1 cm thick. Over the 24 h incubation, we observed the accumulation of bicarbonate-derived C in the bottom lamina (up to 1 cm depth; Figure 2), which is an area always below the photic zone. Given this long transfer distance, it is unlikely that cell migration alone was responsible for the movement of fixed C over the diel cycle. This conclusion is supported by comparing both the distinction and stability of LA-IRMS profiles in the acetate and glucose incubations which showed much less vertical migration of incorporated C vs. the bicarbonate incubations. Taken together, our result suggests that transfer of fixed C from photoautotrophs to heterotrophs in this microbial mat ecosystem takes place via the transport of aqueous organic compounds from higher to lower laminae within the system, via metabolites, EPS, and/or necromass. Our results are consistent with previous work which suggested small organic molecules (included in EPS) could be viewed as a community resource in cyanobacterial-driven hypersaline microbial mats, with competition for these substrates helping to fuel their rapid turnover and recycling (Stuart et al., 2016a,b; Gonzalez-Nayeck et al., 2022). Building upon this interpretation, we demonstrated that in addition to these C exchange processes directing rapid C exchange between functional guilds, they also facilitate spatial redistribution of freshly fixed organics throughout the microbial mat profile and likely play a central role in maintaining the viability of mat layers consistently or temporarily (i.e., during the diel) below the photic zone.

## 5. Conclusion

Our integration of spatial, biochemical, and taxonomic tracking of substrate incorporation into a laminated microbial mat allowed elucidation of the rapid metabolic interconnections involved in C exchange between photoautotrophic and heterotrophic members. Bicarbonate-derived C was initially focused within the photic zone of the mat but then distributed vertically through the mat within hours during daylight periods. The spatial migration of bicarbonate-derived C into greater depths within the mat profile is consistent with high rates of C exchange between photoautotrophs and consumers as documented by ^13^C labeled protein expression. Incorporation of ^13^C from labeled glucose and acetate displayed less vertical migration and less diel control on uptake and exchange of substrate-derived C. The time-dependent, as opposed to diel-dependent, uptake of glucose and acetate into specific proteins indicates most mat organisms, including cyanobacteria, are primed to use organic C sources.

## Data availability statement

The datasets presented in this study can be found in online repositories. The names of the repository/repositories and accession number(s) can be found in the article/Supplementary material.

## Author contributions

JJM conceived of the study, oversaw data collection, and wrote the manuscript. HCB guided interpretation of LA-IRMS data and wrote the manuscript. ABC, SC, and JJM conducted the field experimentation and sample collection. Y-MK led and performed the metabolite analysis and interpretation. JMM, AMT, and MSL conducted the proteomic analysis and data interpretation. KLD, ABC, and JJM completed the stable isotope measurements and interpretation. RSR performed the NMR sample analysis and interpretations. JKF and HWK provided the assistance with experimental design, data analysis, and manuscript development. All authors contributed to the manuscript and approved the submitted version.

## Supplementary material

Supplementary Figure 1 Experimental samples, incubation apparatus, and sampling timepoints. Samples **(A)** were collected from Hot Lake. The benthic phototrophic communities were roughly one cm in thickness and harvested as a cohesive unit and then divided between incubation containers (inset shows). The incubations were performed in two individual, insulated boxes. The first **(B)** was covered with clear acrylic sheets which were opened or closed during the day to moderate temperature while the second box was very similar but had an opaque lid to exclude light. Hot water (collected from at depth in the lake) and hot packs were used to maintain temperature during the night. Irradiance measurements **(C)** corresponding to each sampling time point were used to estimate the photic depth within the samples following the Beer-Lambert relationship.

Supplementary Figure 2 2D porosity map of mat using chemical shift selective imaging. A small syringe can be seen near the top of the mat, which was used to supply 100 mM acetate. Acetate was tracked over time and the mat was fully saturated within 7 h of application.

Supplementary Figure 3 Identification of 13C uptake into abundant metabolites. Mass spectral data are shown for abundant metabolites where response is normalized to the molecular ion abundance. Label incorporation is observed in cases where the relative abundance of a mass is elevated compared to the control (incubations with unlabeled bicarbonate additions). Monitored ions; glucose (319 m/z), sucrose (361 m/z), trehalose (361 m/z), glucosylglycerol (361 m/z), 3-hydroxybutanoic acid (233 m/z), and 3-hydroxypentanoic acid (247 m/z).
