# Supplementary material for: Daylight-driven carbon exchange through a vertically structured microbial community
Source: Front Microbiol. 2023 May 26;14:1139213. doi: 10.3389/fmicb.2023.1139213 (PMC10251406; doi:10.3389/fmicb.2023.1139213)
Supplement: Supplementary file 5 [file Data_Sheet_1.pdf]

## SUPPLEMENTAL EXPERIMENTAL PROCEEDURES

**Estimating replacement of biomass by substrate C.** We used an isotopic mass balance approach to estimate the amount of C, at each laser ablation sampling location, that had been replaced during the experiment with C originating from the added substrate in each incubation (bicarbonate, glucose, or acetate). We first converted the measured $\delta^{13}C$ value for each laser ablation sample pit into its associated isotopic ratio ($R = {}^{13}C/{}^{12}C$) using equation S1:

$$R_{sample} = \left(\frac{\partial^{13}C}{1000} + 1\right) R_{VPDB} \qquad \text{Equation S1}$$

where $RV_{PDB}$ is the isotopic ratio for the internationally recognized isotope standard (Vienna Pee Dee Belemnite, 0.0112372). The isotopic ratio (R) is then converted to fractional abundance ($F = {}^{13}C$/Total C) using equation S2:

$$F = \frac{R}{1+R} \qquad \text{Equation S2}$$

Finally, a weighted mass balance approach, derived from Equation S3, is used to quantify carbon conversion from substrate into mat biomass.

$$F_{measured} = xF_{control} + (1-x)F_{substrate} \qquad \text{Equation S3}$$

where x is the fractional abundance of initial biomass (not containing carbon derived from the added substrate) and (1-x) represents the proportion of biomass derived from the added substrate.

We calculated biomass replacement in the acetate and glucose incubations under the assumption there was very minor native acetate and glucose in the system to enable $F_{substrate}$ to approach 1 (given we used 99+ % $^{13}C$ labelled substrates). Due to this assumption, our estimates for the conversion of acetate- and glucose-derived C into biomass represent a minimum amount where the increased presence of native (unlabelled) acetate and glucose would increasingly cause us to underestimate conversion to biomass. The high salinity content of this system precluded typical analytical approaches for estimating native acetate and glucose concentrations. Within the bicarbonate incubations, however, there was appreciable native bicarbonate (10.63 mM C with $\delta^{13}C$ = -2.12 ‰ as measured by Colorado Plateau Analytical Laboratory, Flagstaff, Arizona, USA) in the system. In this case we used a weighted mass balance of the native and added bicarbonate to calculate the total $F_{substrate}$ (0.0959131) before applying equation S3.
