# Supplementary figures and images for: Daylight-driven carbon exchange through a vertically structured microbial community
Source: Front Microbiol. 2023 May 26;14:1139213. doi: 10.3389/fmicb.2023.1139213 (PMC10251406; doi:10.3389/fmicb.2023.1139213)

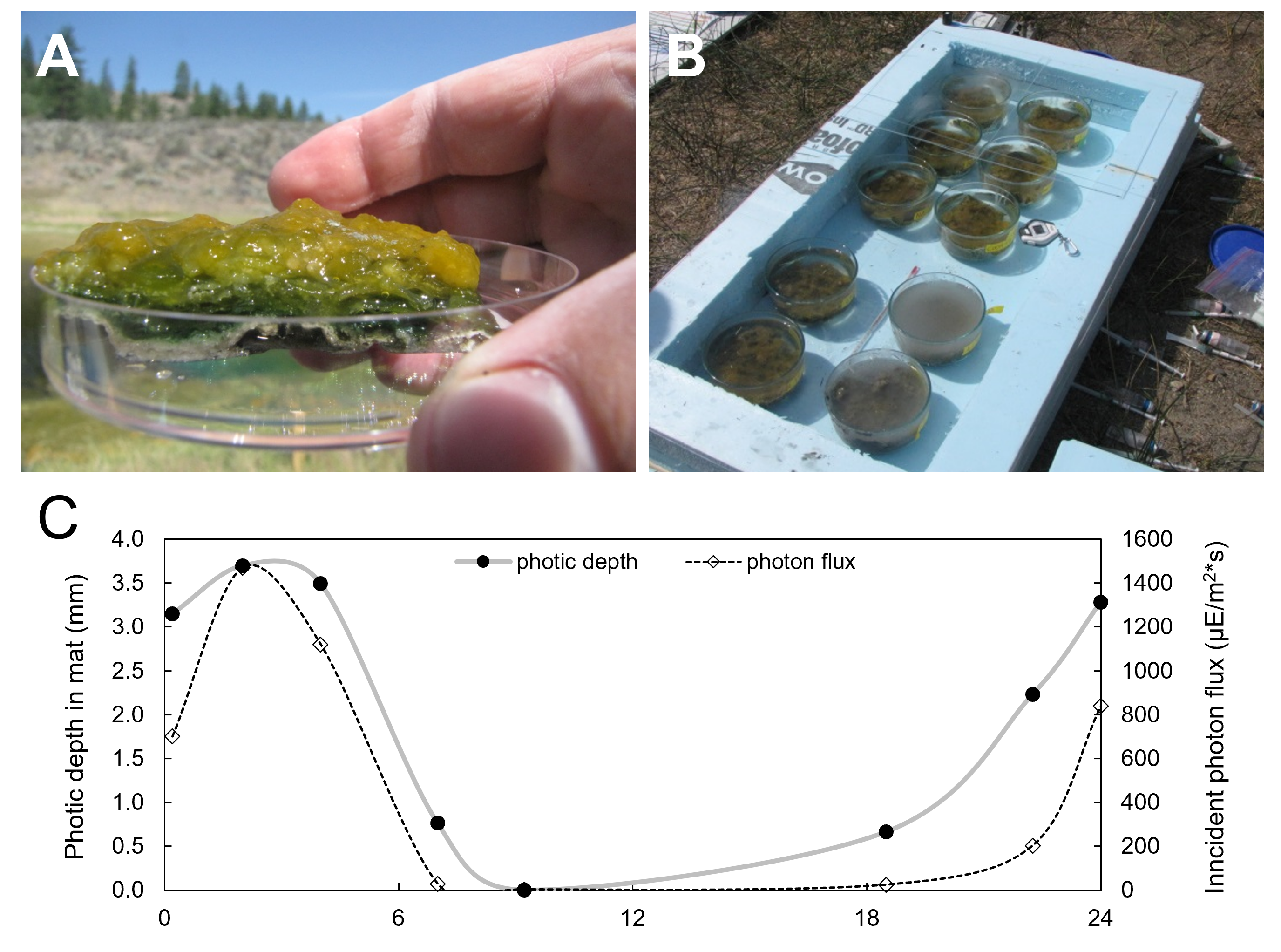

Supplement: Supplementary Figure 1 — Experimental samples, incubation apparatus, and sampling timepoints. Samples (A) were collected from Hot Lake. The benthic phototrophic communities were roughly one cm in thickness and harvested as a cohesive unit and then divided between incubation containers (inset shows). The incubations were performed in two individual, insulated boxes. The first (B) was covered with clear acrylic sheets which were opened or closed during the day to moderate temperature while the second box was very similar but had an opaque lid to exclude light. Hot water (collected from at depth in the lake) and hot packs were used to maintain temperature during the night. Irradiance measurements (C) corresponding to each sampling time point were used to estimate the photic depth within the samples following the Beer-Lambert relationship. [file Image_1.TIF]

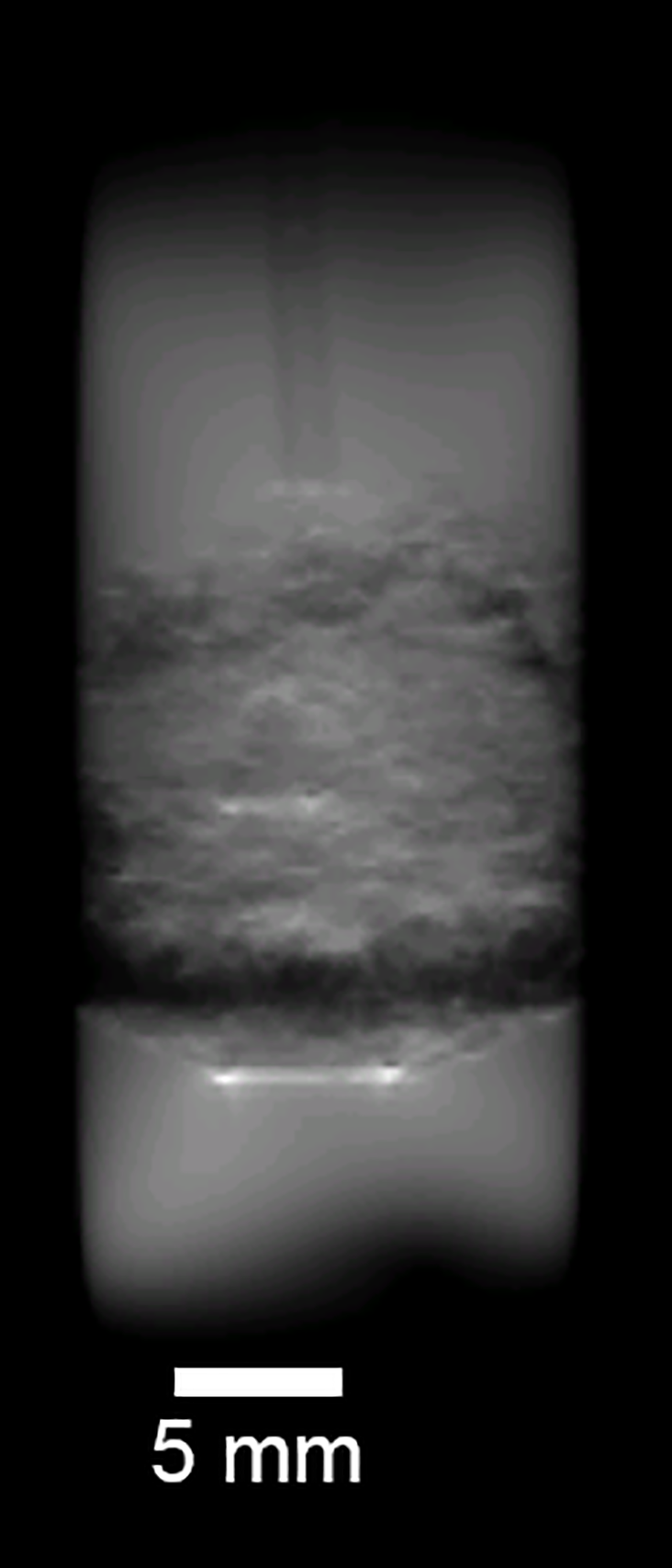

Supplement: Supplementary Figure 2 — 2D porosity map of mat using chemical shift selective imaging. A small syringe can be seen near the top of the mat, which was used to supply 100 mM acetate. Acetate was tracked over time and the mat was fully saturated within 7 h of application. [file Image_2.TIF]

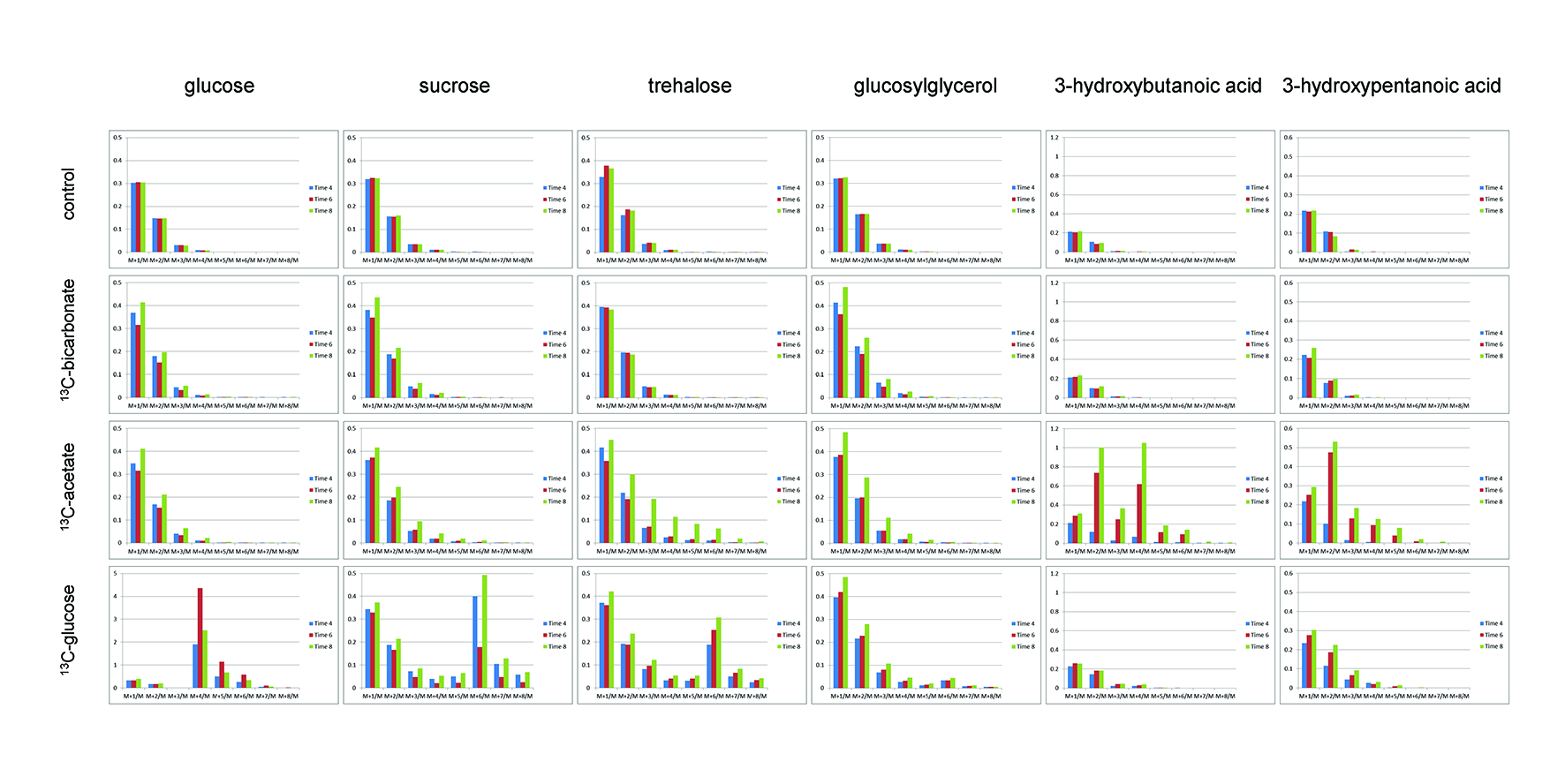

Supplement: Supplementary Figure 3 — Identification of 13C uptake into abundant metabolites. Mass spectral data are shown for abundant metabolites where response is normalized to the molecular ion abundance. Label incorporation is observed in cases where the relative abundance of a mass is elevated compared to the control (incubations with unlabeled bicarbonate additions). Monitored ions; glucose (319 m/z), sucrose (361 m/z), trehalose (361 m/z), glucosylglycerol (361 m/z), 3-hydroxybutanoic acid (233 m/z), and 3-hydroxypentanoic acid (247 m/z). [file Image_3.TIFF]
